# Comment on “Promising blood-derived biomarkers for estimation of the postmortem interval” by I. Costa, F. Carvalho, T. Magalhães, P. G. de Pinho, R. Silvestre & R. J. Dinis-Oliveira. (*Toxicol. Res.*, 2015, **4**, 1443–1452)

**DOI:** 10.1039/c5tx00397k

**Published:** 2016-02-02

**Authors:** Joris Meurs, Katarzyna M. Szykuła

**Affiliations:** a VU University Amsterdam , Faculty of Sciences , Amsterdam , The Netherlands . Email: j.meurs@student.vu.nl; b University of Amsterdam , Faculty of Sciences , Amsterdam , The Netherlands

## Abstract

Recently, Costa *et al.* published an article about promising biomarkers for estimating the postmortem interval. Instead of postmortem blood, antemortem blood was putrefied *in vitro* by exposing the blood to a temperature gradient. However, in this way several other influencing factors were excluded, hence, the accuracy of the proposed model is doubtful. Therefore, the aim of this comment is to discuss the methodology, results and shortcomings of the study of Costa *et al.*

## Abstract 2

Recently, Costa *et al.* published an article about promising biomarkers for estimating the postmortem interval.

## 1. Introduction

Postmortem interval (PMI) estimation has always been a major interest in forensic and legal medicine.^1^ Classical methods such as *algor mortis*, *livor mortis* and *rigor mortis* are still widely used for estimating the PMI, though, these methods have a relatively large error.^2^–^4^ Therefore, research has focused on alternative methods such as biochemical markers.^5^

Recently, Costa *et al.* published an article on promising blood-derived biochemical markers.^6^ In this article, a new model was proposed for PMI estimation. However, this model is based on the results of mimicked postmortem blood samples. These samples were exposed to a simulated cooling process and concentrations of numerous analytes were measured at several time intervals. At this stage of the study it is too early to propose a model for PMI estimation. Therefore, the opportunity was taken to comment on experimental design and shortcomings of Costa *et al.*^6^

## 2. Comments

First of all, no correlation is shown between the concentration in real postmortem blood samples and the blood samples analyzed in this study. Hence, it cannot be concluded if the measured concentrations are reflecting the concentrations in postmortem blood. We made a comparison between the compounds used for the proposed model by Costa *et al.* and studies which investigated the same compounds in postmortem serum. Conflicting results were found for urea,^7^ uric acid,^7^ total bilirubin,^8^ calcium^9^ and creatine kinase MB.^10^ No significant alteration was observed for urea, uric acid, total bilirubin and calcium,^7^–^9^ while Costa *et al.* observed significant alterations for these compounds.^6^ In case of creatine kinase MB, the correlation coefficient varied between 0.266 and 0.304,^10^ which is much lower than the threshold of 0.900 in the commented article. Due to this conflicting results the reliability of this model is doubtful. Therefore, it is too optimistic to claim that the model gives an accurate estimation of the PMI.

Secondly, the question arises to what extent postmortem blood can be mimicked by exposing antemortem blood to simulated postmortem conditions. In this study, only the temperature was chosen as influencing factor. Consequently, other important factors were excluded as: gender, age, biological background, lifestyle, cause of death, sample site, postmortem redistribution, and other environmental conditions.^5^,^11^,^12^ These factors should be taken into account as was shown for instance by Palmiere & Mangin.^7^ They observed that the concentrations of uric acid and urea are gender-dependent.^7^ Further, we noticed that Costa *et al.* made several comparisons between their results and real postmortem blood. In our opinion, these comparisons cannot be made as foregoing factors were excluded in their study. Furthermore, a hypothesis was made about the alteration of the pH *in situ* and *in vitro*. It is mentioned by Costa *et al.* that the pH is changing due to the release of compounds from putrefying organs.^6^ Thus, according to their hypothesis the alteration of pH depends on *in situ* processes. However, the emphasis on the differences between *in situ* putrefaction and *in vivo* putrefaction and the link to the reliability of the model is missing in our opinion.

Thirdly, Costa *et al.* investigated venous blood up to 264 hours (11 days). However, obtaining postmortem venous blood becomes more difficult at increasing PMI due to the probability of postmortem blood clotting in the first hours after death.^13^,^14^ In addition, postmortem lividity will lead to sedimentation of blood in the body.^13^–^15^ Hence, the amount of available blood in veins will decrease when the PMI is increasing. Sampling at other sites of the body can be problematic, because the concentration of a certain analyte can differ significantly per sample site.^16^ According to this, the time interval of sampling is too large compared to the practical time interval of sampling dead bodies in our opinion.

## 3. Conclusion

The aim of this comment was to discuss the methodology, results and shortcomings of Costa *et al.*^6^ Recommendations and drawbacks should be more extensively discussed in this article. Additionally, it should be considered to what extent the research can be translated into practice. Furthermore, additional research has to be performed to obtain all necessary data about the influence of internal and external factors. Thereafter, a model for PMI estimation can be proposed. In addition, it has to be considered if sampling of antemortem blood exposed to simulated postmortem conditions should be performed over a large PMI due to the high probability of practical limitations of sampling postmortem blood. Therefore, we question the reliability of using *in vitro* studies to draw conclusions about postmortem changes due to conflicting results between *in vitro* and *in vivo* studies.

## Conflict of interest

None.
